# Do cognitive and non-cognitive abilities mediate the relationship between air pollution exposure and mental health?

**DOI:** 10.1371/journal.pone.0223353

**Published:** 2019-10-23

**Authors:** Ting Ren, Xinguo Yu, Weiwei Yang

**Affiliations:** HSBC Business School, Peking University, Shenzhen, Guangdong, China; University of Cape Coast, GHANA

## Abstract

Considered as a key component of human capital, mental health has drawn substantial scholarly attention for its effect on people’s health status and economic outcome. When facing the challenge of stress, people’s heterogeneity in cognitive ability and non-cognitive ability causes difference in patterns of coping, resulting in different manifestations in mental health. Previous researches have shown that cognitive and non-cognitive abilities have positively direct or indirect effects on mental health, but few studies research their role of coping with air pollution. We used the China Family Panel Survey (CFPS) and matched individual data with county or district level PM_2.5_ information from NASA. The study found that air pollution has negative effect on mental health with every increase of 1μg/m^3^ in PM_2.5_ deteriorating mental health by 0.038 standard deviation, which is the total effect of air pollution. However, the direct effect of air pollution on mental health will decrease to 0.028 in absolute value when considering mediating effects. By employing different approaches, we found positive mediating effects via cognitive ability and non-cognitive ability. Individuals with high cognitive and non-cognitive abilities are able to accurately diagnose problems and select the optimal coping strategies, thus restoring positive mental health.

## Introduction

**Fig 1:**
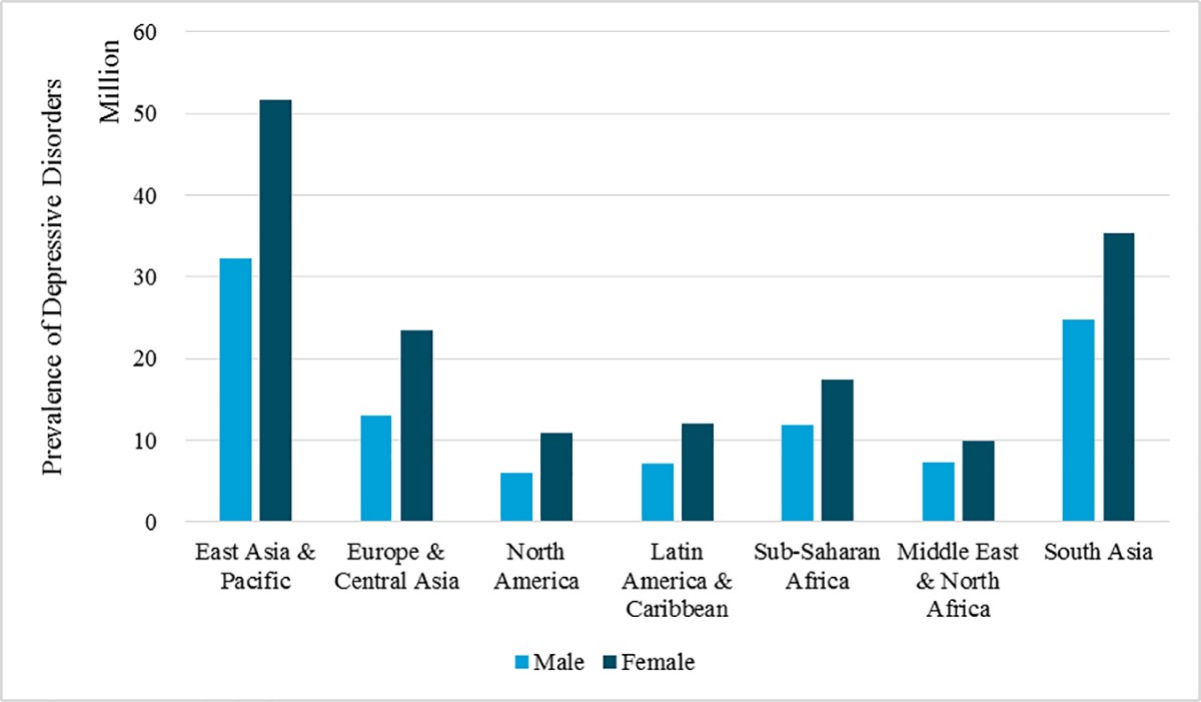
Global Prevalence of depressive disorders, by region and gender in 2017 source: Global burden of disease study 2017 (http://ghdx.healthdata.org/gbd-results-tool).

Mental health can be a global matter, and so common that people across ages, jobs, regions might all suffer from mental illnesses. According to the World Health Organization, the two most common mental disorders were depression and anxiety disorders, and these two mental disorders together caused up to one billion U.S. dollars annual global productivity loss; more than 322 million people (4.4% of the global population) were suffering from depression, making it the worldwide most epidemic disease; and more than 264 million (3.4% of global population) were suffering from anxiety disorders, whose number of patients ranked sixth among all diseases [1]. Fig 1 shows the global prevalence of depression across regions and genders in 2017, according to Global Burden of Disease Study. From 1990 to 2017, depressive disorders developed worsen trend and became the tenth leading cause attributing to disability-adjusted life years for Chinese [2].

Developed countries and developing countries performed quite differently in the prevalence of mental disorders. Some researchers used nationally representative panel data in developed country to reveal the relationship between air pollution exposure and psychological distress [3]. But these mental illnesses could be more common among people who were poor, unemployed, and elderly, especially in the developing countries with a large group of low-income population and growing social inequality in health. Some evidence shows that in India or southern Asia, ambient PM2.5 ranks among the largest risk factors for health loss and premature death [4]. Defensive expenditures against air pollution increased in developed countries while the willingness to invest in private sector of protective tool production show disparity in China [5–8]. Hence, population were more vulnerable to depression and anxiety, making the number of people in developing countries suffering from mental illnesses have grown even faster than in developed countries, which drawn our attention on this problem in China.

Mental health was worth a thorough study for its close association with work productivity and life satisfaction. Numerous studies have shown that poor mental health had a negative impact on personal performance and employees’ productivity [9–11]. As a component of human capital, people’s mental health status could significantly affect their work efficiency and effectiveness, which in turn affected organizational performance. Furthermore, mental health could be a core dimension in life to love and be loved, to work happily and to live happily, and to expect or hope for the future [12]. Therefore, health element of human capital should be considered as an engine for production and the role of environmental pollution should not be underrated [9].

## Literature and hypotheses

Studies about adverse effects of air pollution on health, especially physical health, have emerged in recent decades. Exposure to pollutants such as ambient participate matter and ozone was associated with increases in mortality and hospital admissions due to respiratory and cardiovascular diseases [13]. Research has uncovered that air pollution had significant effects on incidence of cardiac arrhythmia [14], non-malignant respiratory deaths [15], lung cancer mortality [16], asthma mortality [17,18], non-malignant cardiopulmonary deaths [19], and total mortality [20–22], thus shortening life expectancy [16]. Especially individuals exposed to particulate matter pollution were potentially more vulnerable to cardiovascular disease [23]. The mechanisms could be that air pollutants, including ozone, oxides of nitrogen, and suspended particulates, being potent oxidants, exerted toxic effects on the respiratory and cardiovascular systems either through direct effects on lipids and proteins or indirectly through the activation of intracellular oxidant pathways [24].

Compared to an abundance of researches studying the effects of air pollution on physical health, literature focusing on the influence of air pollution on mental health may be limited. Extant studies suggested that air pollution had significantly negative effect on mental health. Nitrogen oxides together with other annoying environmental factors were positively related to anxiety risk [25]. Some pollutants might increase depressive symptoms among the elderly [26]. Particularly, some researchers found exposure to ambient fine particulate matter pollution would increase the risk of depressive symptoms or worsen mental health in low and middle-income countries [27,28]. By investigating the nervous system response to the environmental variations, researchers showed that the exposure to particulate matter pollution had acute effects on hospitalization for mental disorders in China [29]. Air pollution even deteriorated potential psychiatric disorder and mental distress, increasing depression risk in the general population [30,31]. Some scholars suggested that various air pollutants were associated with mental disorders by the toxicity on central nervous system [32].

The optimal state of mental health might be sought after by everyone; however, mental health could be closely related to not only personal traits such as thinking mode or emotion control ability, but also external conditions such as social, cultural, economic, political and environmental conditions [33]. Under the same external conditions, the individual’s mental health may not be alike due to differences in individual traits, such as cognitive ability, which could be represented as synthesis of memory, information processing ability, intelligence, word knowledge, mathematical reasoning, motor skill and problem solving ability [34–38], and non-cognitive ability, which could be defined as the sum of all the stable dimensions of personality, locus of control, self-esteem, curiosity, motivation, responsibility, perseverance, social skills and other traits [36,37,39–41].

When facing environmental stress and pollution, individual heterogeneity in cognitive ability and non-cognitive ability could cause difference in patterns of coping, resulting in different manifestations in mental health. Individual subjective diagnosis and assessment of the problem would determine the coping strategies with the mental problem [42,43]. Individual daily activities and social behaviors are influenced by air pollution. Some researchers found smog alerted individual outdoor activities mode [44,45]. Air pollution was associated with increased defensive expenditures in private protective tools in China [5–8]. Personal traits such as degree of responsibility, the ability of self-control, eagerness to be recognized and other non-cognitive abilities could affect the individual’s impression of change and perception of stressors [46].

Air pollution and other environmental stress could influence the role of cognitive and non-cognitive abilities on mental health. Under risks and pressures, the differences in individuals’ cognitive and non-cognitive abilities could result in diverse subjective judgments and analyses, which further led to distinct ways of response and coping strategies. Cederblad et al. [12] suggested that intelligence, perseverance, reliability, controls of impulses, kindness and other personality traits were protective factors that promoted positive mental health. Cognitive ability and non-cognitive ability were positively associated with labor market outcomes that positively affected mental health [47–49].

Our study focused on the relationship among air pollution, mental health, cognitive ability and non-cognitive ability. By using the micro data of individual traits and air pollution exposure, we explored the influence of air pollution on mental health, and the role played by cognitive and non-cognitive abilities in the relationship between air pollution and mental health. Consistent with the previous arguments [50,51], we found that cognitive ability and non-cognitive ability contributed to maintaining positive mental health. Moreover, these two abilities could mediate the influence of air pollution on mental health.

From the related literature, pollution exposure had negative impacts on the short-term and long-run health status [5,52,53]. Air pollution also reduced subjective well-being, caused anxiety and depression, and seriously increased suicide risk among people exposed to air pollution [25,26]. The mechanism through which air pollution damaged mental health could be that air pollutants were neurotoxic, and people’s neural system would be harmed when exposed to these pollutants. Long-term exposure to ambient particulate matter and ozone caused decline in motor response speed to a visual stimulus, coding ability, attention and short-term memory [54]. Some nationally representative studies in low and middle-income or developed country revealed negative impact of air pollution exposure on psychological distress [3,27,28]. When using a holistic measurement such as Air Pollution Indicator, empirical results supported that air pollution could impair global cognition, ability to perform everyday activities, and satisfaction with health [55,56]. The mechanism could be the response to the environmental variations in nervous system biologically, which the air pollutants increased mental distress by different etiology and toxicity [29,30,32]. Considering the harm of air pollution to mental health from similar researches on China context [28,29,38,57], we proposed the hypothesis about the effect of air pollution:

*Hypothesis 1*: *Air pollution has a negative influence on mental health*.

Empirical studies also supported that cognitive ability, measured by IQ, math test scores and literacy test scores, positively influenced mental health [12,48]. But air pollution also damaged cognitive capability. It directly affected decision making by damaging cognitive ability strategic thinking [58]. Evidence showed air pollution exposure would harmfully affect cognitive ability in academic performance [59]. Short-term memory, mathematical reasoning and such cognitive abilities would be harmed by air pollution [53], which was especially the case for the middle-aged and the elderly [53,60]. Also long exposure to black carbon, a marker of traffic-related air pollution, could cause decreased cognitive function in older men, such as attention, working memory, long-term verbal memory, visuoconstruction and global cognition [61].

There was positive correlation between exposure to fine particulate matter and mild cognitive impairment which was associated with a high risk of progression to Alzheimer's disease in older women by damaging their cognitive abilities of attention, information processing, confrontation naming, executive functions, verbal memory, nonverbal memory, visuoconstruction, global cognition, and olfactory function [62,63]. What’s more, cognitive ability had indirectly positive influence on mental health by affecting academic achievement, personal wages, and employment or reducing drug use, alcoholism, and risky social behavior such as teenage pregnancy and criminal activity [47–49].

The mechanism through which air pollution affected cognitive ability was that air pollution, as a threat to health, could result in decreased adjustment function of cognitive ability on mental health. Through accurate appraisal, selecting the optimal coping strategies, and better implementation of coping strategies, cognitive abilities could be the protective factors of mental health to had better diagnosis of problem, perceiving situations accurately as challenge or threat:

*Hypothesis 2*: *The effect of air pollution on mental health is mediated by cognitive ability*.

As for the mechanism of non-cognitive ability, empirical researches supported that, factors such as perseverance, locus of control, and self-esteem, had positive influence on mental health [12,48]. Through positive appraisal and successfully seeking out social support, people had less exposure to stressor and more effectiveness of coping response. Also non-cognitive ability had indirectly positive influence on mental health by affecting academic achievement and labor market outcomes, or social skills and personality traits were complementarities [37,41,47–49]. According to the perspective of person-environment fit, individuals preferred environments consistent with their own values and beliefs, thus they would strive to solve problems and make adjustments in an effort to adapt to the environmental change [64].

Non-cognitive ability partly determined the capacity to cope with environment effect on mental health across individual endowments [65]. Some mechanism could be reducing people’s motivation to go out and reducing people’s daily outdoor activities, limiting communication and social interaction that in turn led to negative emotions [66,67]. So individuals would diagnose problems and react in diverse manners; for the existence of human heterogeneity, especially in individual’s non-cognitive ability, it would lead to difference of the individual’s subjective judgment:

*Hypothesis 3*: *The effect of air pollution on mental health is mediated by non-cognitive ability*.

## Methodology

### Data and measures

We utilized China Family Panel Study (CFPS) data matched with county or district level data on fine particular matter (PM_2.5_) to test the influence of air pollution on people’s mental health, and mediated by cognitive ability and non-cognitive ability. CFPS was initiated by Peking University and carried out by the Institute of Social Science Survey, with individual, household and community data collected every two years. Starting from 2010 and until 2016, it was a nationally representative and longitudinal survey of general individual economic, social, culture and health status, covering twenty-five provinces in China.

Our research utilized the data of concentration for PM_2.5_ collected from The Global Annual PM_2.5_ Grids provided by U.S. National Aeronautics and Space Administration open source Socioeconomic Data and Applications Center (SEDAC). The database starting from 1998 to 2016 measured the near-surface PM_2.5_ concentration (μg/m^3^), with dust and sea salt removed [68]. NASA’s dataset were gridded into 0.01 degree (1.1 kilometers); therefore, we agglomerated the 0.01 degree grid cells by county or district. The PM_2.5_ concentration values of each 0.01 degree grid cell were aggregated into district or county level values. Take Pudong New District, Shanghai in 2016 for instance, there were 608 grid cells in total, each with a value of annual PM_2.5_ concentration, ranging from a minimum level of 42.00 μg/m^3^ to a maximum level of 51.90 μg/m^3^, with a standard deviation of 2.02. We employed the arithmetic mean of annual PM_2.5_ concentration of all these grid cells, which was 48.41 μg/m^3^, to represent the degree of air pollution.

*Mental health* employed two measurements in previous literature. The first was the diagnosis of specific mental illnesses through interviews and surveys that were widely used by Composite International Diagnostic Interview and its various revised versions. The second method was to measure the psychological pressure in a broad range, rather than diagnose specific mental illness through specific symptoms. The best example was General Health Questionnaire (GHQ) by Goldberg [69] and Mental Health Inventory (MHI-5) by Veit and Ware [70]. Das et al. [71] used 20 self-reported questions about depression that required respondent to answer the frequency of a mental state occurrence within the last month, such as the frequency of feeling depressed, nervous and anxious, unable to sleep, and unable to concentrate on doing things.

The measurement of mental health used in this study was similar to Das et al. [71], containing a series of questions about the frequency of depression. The 2016 data on mental health was measured by the Center for Epidemiological Studies Depression Scale (CES-D), while the 2014 data on mental health was measured by the Kessler Screening Scale for Psychological Distress (K6). We used Principal Component Analysis (PCA) to synthesize these sub-dimensions to proxy mental health. In social surveys, when a series of questions were used to measure a person’s opinion, PCA could be used to construct one representative measurement.

CES-D in 2016 was composed of six scales: depression (the frequency of feeling depressed), difficulty (the frequency of feeling that everything was difficult), poor sleeping (the frequency of poor sleeping), loneliness (the frequency of feeling lonely), sadness (the frequency of feeling sad), and hardness (the frequency of that life was hard to go on). Each scale ranged from 1 to 4, standing for almost never (less than 1 day per week), sometimes (1 to 2 days per week), often (3 to 4 days per week), and almost daily (5 to 7 days per week). The six scales of K6 in 2014 included depression (the frequency of feeling depressed), nervousness (the frequency of feeling nervous), restlessness or fidgetiness (the frequency of feeling restless or fidgety), hopelessness (the frequency of feeling hopeless), difficulty (the frequency of feeling that everything was difficult), and worthlessness (the frequency of feeling that life was worthless), each of them ranging from 1 to 5 according to the frequency.

*Cognitive ability* in related studies commonly used test scores of reasoning, vocabulary, comprehension, mathematics, and coding speed [36,47,60]. Many empirical studies on cognitive ability used math and verbal scores as measurement of cognitive abilities such as memory, information processing and mathematical reasoning. Then cognitive ability used in our study could be suggested as stable and mature instruments. It composed of the score of immediate word record test score and number series test score in 2016, and composed of word test score and math test score in 2014.

*Non-cognitive ability* in most literature used self-reported scale of personality, locus of control, self-esteem, leadership, interpersonal communication and social skills as measurements [36,37,39,41,48,49]. But self-reported measurements had problems with inaccurate and unstable estimates in social skills. More stable and valid data were added with the interviewers’ observation in our study. Non-cognitive ability was composed of the ability to express oneself and the extent of Mandarin fluency under interpersonal communication, both rated by the interviewers. The logic was that communication competence, or language usage and expression fluency were significantly and positively correlated with interpersonal relationship as well as cultural and environmental adaptation [72].

*Covariates* in the literature on mental health included the demographic characteristics, social characteristics, and economic characteristics of individuals. According to Das et al. [73], Das et al. [71] and Zhang et al. [38], factors that affected mental health included demographic characteristics, social characteristics, and economic characteristics such as age, gender, education attainment, marital status, and physical health. Covariates in this study included relative income level, social status, age, gender, and the level of education obtained, marital status. Relative income level in the local area was scored in the range of 1–5 from the low to the high; social status was determined by the respondent’s status in the local area, scored in the range of 1–5. Gender was a dummy variable, with the value of 1 standing for male and 0 for female. Marital status included the status of unmarried, cohabitation, divorce, and widowhood, which were all dummies. The level of education attainment included illiterate or semi-literate, primary schools, junior high schools, senior high schools, 3-year colleges, undergraduates, masters, and all were dummy variables.

### Empirical model

**Fig 2:**
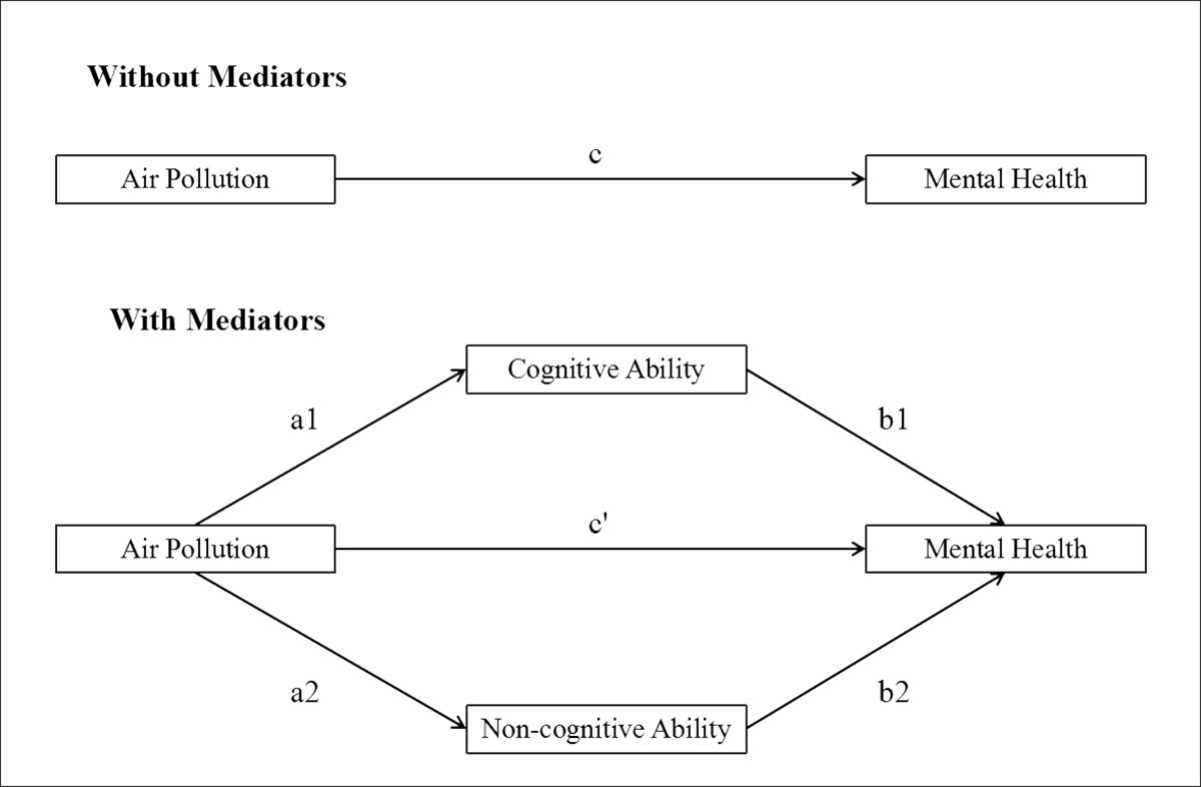
The influence of air pollution on mental health, mediated by cognitive ability and non-cognitive ability.

In order to investigate the influence of air pollution on mental health as well as the mediating effects of cognitive and non-cognitive ability, we used path analysis and Monte Carlo approach to developed research structure. The conceptual framework was shown in Fig 2 as follows:

The framework above described the relationship between air pollution and mental health as well as the mediating effects via cognitive ability and non-cognitive ability. The first was the model without mediators, and path ***c*** was the ***total effect***. The second was the model with mediators, suggesting the effect of air pollution on mental health was mediated by cognitive ability and non-cognitive ability. Path ***c’*** was the ***direct effect***. Both path a1*b1 and path a2*b2 were ***indirect effects***, specifically, path a1*b1 was the indirect effect via cognitive ability and path a2*b2 was the indirect effect via non-cognitive ability. Path a1*b1+ a2*b2 was ***total indirect effects***. It was suggested that the structure equations should be used and contain the following models:

1. Model without Mediators:
  MentalHealth=c*AirPollution+Covariates+ErrorTerm
2. Effect of Air Pollution on Cognitive Ability:
  CognitiveAbility=a1*AirPollution+Covariates+ErrorTerm
3. The Effect of Air Pollution on Non-cognitive Ability:
  Non‐cognitiveAbility=a2*AirPollution+Covariates+ErrorTerm
4. Model with Mediators:
  $$\mathrm{Mental}\;\mathrm{Health}=c^{\prime }*\mathrm{Air}\;\mathrm{Pollution}+b1*\mathrm{Cognitive}\;\mathrm{Ability}+b2*\mathrm{Non}‐\mathrm{cognitive}\;\mathrm{Ability}+\mathrm{Error}\;\mathrm{Term}$$

## Results

### Descriptive analysis

We employed the most recent data in 2016 from CFPS adult survey. Then we used the 2014 data to test whether the results using the 2016 data was reliable. The adult data in 2016 wave were collected from 33,296 individuals above 16 years old, with 50% for both female and male. 48.93% were from urban, 49.93% were from rural area, and 1.14% with missing values. This database in 2014 wave collected information from 37,147 individuals aged above 16, with 50% female and 50% male. Among these interviewees, 44.1% were from urban and 48.14% were from rural area, leaving the rest 7.85% unrecognized.

In this study, we first dropped data with missing values on dependent variable, independent variables and covariates. Secondly, we matched the individual data from CFPS with county or district level data on PM_2.5_. Finally, 13,761 observations were obtained in 2016, and 18,229 observations in 2014. From Table 1 above, in our sample of 2016, the 13,761 observations were equally distributed for both male (49.42%) and female (50.58%), as well as for both urban area (48.48%) and rural area (51.52%). In the sample of 2014, the 18,229 observations were equally distributed for both male (51.46%) and female (48.54%), and almost equally distributed for both urban area (51.35%) and rural area (48.65%). For both cross-sections, depression, cognitive ability, as well as non-cognitive ability were standard normal distributions. PM_2.5_ ranged from 2.64 μg/m^3^ to 80.97 μg/m^3^ in 2016, with a mean of 38.625 μg/m^3^ which was higher than the safe level of 25 μg/m^3^ according to WHO.

**Table 1:** Statistical description†.

	2016					2014				
Variable	Obs	Mean	Std. Dev.	Min	Max	Obs	Mean	Std. Dev.	Min	Max
Depression	13761	0	1	-0.989	4.997	18229	0	1	-0.761	5.509
Cognitive ability	13761	0	1	-2.487	3.004	18229	0	1	-1.687	2.174
Non-cognitive ability	13761	0	1	-2.832	1.331	18229	0	1	-2.897	1.408
PM_2.5_	13761	38.625	16.775	2.642	80.97	18229	40.470	15.068	2.952	77.68
Relative Income	13761	2.411	1.009	1	5	18229	2.496	0.974	1	5
Social Status	13761	2.825	1.058	1	5	18229	2.893	0.981	1	5
Age	13761	49.879	15.183	18	98	18229	46.627	15.504	16	95
Gender	13761	0.494	0.500	0	1	18229	0.515	0.500	0	1
Male	6801	(49.42%)				9380	(51.46%)			
Female	6960	(50.58%)				8849	(48.54%)			
Urban	13761	0.481	0.500	0	1	18229	0.505	0.500	0	1
Urban	6672	(48.48%)				9361	(51.35%)			
Rural	7089	(51.52%)				8868	(48.65%)			
Marital	13761	2.111	0.775	1	5	18229	2.090	0.776	1	5
Never married	1111	(8.07%)				1755	(9.63%)			
Married	11646	(84.63%)				15168	(83.21%)			
Cohabitation	77	(0.56%)				101	(0.55%)			
Divorced	219	(1.59%)				315	(1.73%)			
Widowed	708	(5.14%)				890	(4.88%)			
Education Degree	13761	2.564	1.329	1	7	18229	2.706	1.334	1	8
Illiterate	3779	(27.46%)				4107	(22.53%)			
Primary school	3040	(22.09%)				4143	(22.73%)			
Junior high school	3927	(28.54%)				5443	(29.86%)			
Senior high school	1881	(13.67%)				2798	(15.35%)			
3-year college	704	(5.12%)				1059	(5.81%)			
Bachelor's degree	404	(2.94%)				639	(3.51%)			
Master's degree	26	(0.19%)				39	(0.21%)			
Doctoral degree	0	(0%)				1	(0.01%)			

†Notes: Depression is measured by the Center for Epidemiological Studies Depression Scale (CES-D) in 2016 and Kessler Screening Scale for Psychological Distress (K6) in 2014. Percentages of each category of categorical variables are in the parentheses.

### The effect of air pollution on mental health

In this study, we firstly employed the most recent data in 2016 to study the relationship between air pollution and mental health as well as the mediating effects of cognitive ability and non-cognitive ability, and we secondly utilized the second most recent data in 2014 to test the stability and reliability of the results previously obtained from the data in 2016. The results from the 2016 data and 2014 data were presented in Table 2 and Table 3, respectively, but only the results from 2016 data were discussed in details to make the work concise and brief.

**Table 2:** Effect of air pollution on mental health, mediated by cognitive and non-cognitive ability, 2016†.

	Depression	Cognitive ability	Non-cognitive ability	Depression
PM_2.5_	0.038*** (0.015)	-0.059*** (0.011)	-0.052*** (0.011)	0.028* (0.014)
Cognitive ability				-0.112*** (0.011)
Non-cognitive ability				-0.053*** (0.011)
Covariates	Yes	Yes	Yes	Yes
County/District Fixed Effects	Yes	Yes	Yes	Yes
Observations	13761	13761	13761	13761
Adjusted R^2^	0.134	0.461	0.504	0.143

†Notes: Standard errors in parentheses.

* *p* < 0.1

** *p* < 0.05

*** *p* < 0.01. Depression is measured by the Center for Epidemiological Studies Depression Scale (CES-D). PM_2.5_ is a proxy for air pollution. Cognitive ability is composed of the score of immediate word recall test and the score of number series test, and non-cognitive ability is composed of the ability to express oneself (rated by the interviewers) and the extent of fluency of Mandarin (rated by the interviewers). To control for unobserved effects resulting from heterogeneity of different counties or districts which the interviewees live, county or district fixed effects are included in the models. OLS Model is employed in this table. The first column is run with depression regressed on PM_2.5_; the second column with the first mediator (cognitive ability) regressed on PM_2.5_; the third column with the second mediator (non-cognitive ability) regressed on PM_2.5_; and the fourth column with depression regressed on PM_2.5_ and both mediators (cognitive ability and non-cognitive ability).

**Table 3:** Effect of air pollution on mental health, mediated by cognitive and non-cognitive ability, 2014†.

	Depression	Cognitive ability	Non-cognitive ability	Depression
PM_2.5_	0.015** (0.006)	-0.014*** (0.004)	-0.039*** (0.005)	0.012** (0.006)
Cognitive ability				-0.053*** (0.011)
Non-cognitive ability				-0.053*** (0.009)
Covariates	Yes	Yes	Yes	Yes
County/District Fixed Effects	Yes	Yes	Yes	Yes
Observations	18229	18229	18229	18229
Adjusted R^2^	0.101	0.574	0.427	0.104

†Notes: Standard errors in parentheses.

* *p* < 0.1

** *p* < 0.05

*** *p* < 0.01. The explanation of variables and models is the same as those in Table 2, except that mental health is measured by the Kessler Screening Scale for Psychological Distress (K6), and cognitive ability is composed of the score of word test and the score of math test.

To test the effect of air pollution on mental health and check the mediation effect of cognitive ability and non-cognitive ability, we compared two models, one without mediators and the other with mediators. In addition, the pathways from air pollution to the mediators, cognitive ability and non-cognitive ability, were tested. In the model without mediators (the first column in Table 2), the effect of PM_2.5_ on depression was positively significant, with a coefficient of 0.038 at 1% significance level, which means that every increase of 1 μg/m^3^ in air pollution deteriorates mental health by 0.038 standard deviation significantly. PM_2.5_ had significantly negative influence on both cognitive ability (the second column in Table 2) and non-cognitive ability (the third column in Table 2). In the model with mediators (the fourth column in Table 2), the effect of PM_2.5_ on depression was still positively significant, but smaller in magnitude.

There was a concern that cognitive ability was probable to be influenced by mental health, causing endogeneity problem. To control for this endogeneity, we introduced whether or not using Chinese Mandarin (the official language in daily activities) rather than dialects in the interview as an instrumental variable for non-cognitive ability, following Dustmann and Fabbri [74]. The logic was that people who had better performance in non-cognitive ability were more likely to use Mandarin in interview, for the reason that people were considerate for the interviewers and pursue for better communication by using Mandarin, and for the reason that people make efforts to overcome the inclination to use dialects to communicate.

Since the instrumental variable of using Chinese Mandarin in the interview was a dichotomous variable, a Logistic model or a Probit model should be used instead of an OLS (ordinary least square) model. This study employed both the Logistic model and the Probit model when the variable of using Chinese Mandarin in the interview was explained by PM_2.5_.

The coefficient of whether or not using mandarin in the interview explained by non-cognitive ability was statistically significant and positive with a value of 1.293 at 1% significance level, showing that non-cognitive ability was highly correlated with the instrumental variable. Overall, the estimates in the robustness test were consistent with the estimates in the basic results.

When employing a Probit model to predict the effect of non-cognitive ability on Mandarin usage and the effect of PM_2.5_ on Mandarin usage (the sixth to tenth columns in Table 4), the estimates were very similar with the results using Logistic model (see in Table 4). By using data in 2014, we also found the robust results as above in Table 5.

**Table 4:** Robustness model: Effect of air pollution on mental health, mediated by cognitive and non-cognitive ability using logistic/probit model, 2016†.

	Logistic Model					Probit Model				
	IV_Mandarin usage	Depression	Cognitive ability	IV_Mandarin usage	Depression	IV_Mandarin usage	Depression	Cognitive ability	IV_Mandarin usage	Depression
PM_2.5_		0.038*** (0.015)	-0.059*** (0.011)	-0.169*** (0.063)	0.030** (0.015)		0.038*** (0.015)	-0.059*** (0.011)	-0.087*** (0.030)	0.030** (0.015)
Cognitive ability					-0.119*** (0.011)					-0.119*** (0.011)
IV_Mandarin usage					-0.019 (0.025)					-0.019 (0.025)
Non-cognitive ability	1.293*** (0.046)					0.711*** (0.025)				
Covariates	Yes	Yes	Yes	Yes	Yes	Yes	Yes	Yes	Yes	Yes
County/District Fixed Effects	Yes	Yes	Yes	Yes	Yes	Yes	Yes	Yes	Yes	Yes
Observations	10214	13761	13761	10214	13761	10214	13761	13761	10214	13761
Adjusted R^2^		0.134	0.461		0.141		0.134	0.461		0.141
Pseudo R^2^	0.496			0.427		0.495			0.428	

†Notes: Standard errors in parentheses.

* *p* < 0.1

** *p* < 0.05

*** *p* < 0.01. The explanation of variables is the same with those in Table 2. The first and sixth columns are run with the instrument (Mandarin used in interview) regressed on non-cognitive ability; the second and seventh columns with depression regressed on PM_2.5_; the third and eighth columns with the first mediator (cognitive ability) regressed on PM_2.5_; the fourth and ninth columns with the second mediator (Mandarin used in interview) regressed on PM_2.5_; and the fifth and tenth columns with depression regressed on PM_2.5_ and both mediators (cognitive ability and Mandarin used in interview). Both the first and third columns are employing Logistic model, while the sixth and eighth columns are using Probit model.

**Table 5:** Robustness model: Effect of air pollution on mental health, mediated by cognitive and non-cognitive ability using logistic/probit model, 2014†.

	Logistic Model					Probit Model				
	IV_Mandarin usage	Depression	Cognitive ability	IV_Mandarin usage	Depression	IV_Mandarin usage	Depression	Cognitive ability	IV_Mandarin usage	Depression
PM_2.5_		0.015** (0.006)	-0.014*** (0.004)	-0.106 (0.084)	0.014** (0.006)		0.015** (0.006)	-0.014*** (0.004)	-0.042 (0.030)	0.014** (0.006)
Cognitive ability					-0.063*** (0.011)					-0.063*** (0.011)
IV_Mandarin usage					-0.016 (0.021)					-0.016 (0.021)
Non-cognitive ability	1.131*** (0.032)					0.637*** (0.018)				
Covariates	Yes	Yes	Yes	Yes	Yes	Yes	Yes	Yes	Yes	Yes
County/District Fixed Effects	Yes	Yes	Yes	Yes	Yes	Yes	Yes	Yes	Yes	Yes
Observations	15944	18229	18229	15944	18229	15944	18229	18229	15944	18229
Adjusted R^2^		0.101	0.574		0.103		0.101	0.574		0.103
Pseudo R^2^	0.491			0.423		0.490			0.423	

†Notes: Standard errors in parentheses.

* *p* < 0.1

** *p* < 0.05

*** *p* < 0.01. The explanation of variables is the same with those in Table 3, and the explanations of models are the same as those in Table 4.

### The mediating effects of cognitive ability and non-cognitive ability

The mediating part was of interests in most studies when causal or structural models were examined [75], so was the case in this study. In order to understand the mechanism that air pollution influences mental health through cognitive ability and non-cognitive ability, this study demonstrated the mediating effects in different ways. Firstly this study compared between estimates of the model without mediators and the model with mediators, distinguishing the path c, the total effect, from the path c’, the direct effect, so as to see the difference or the indirect effects. The second way to demonstrate the mediating effect was path analysis, which was to check the path a1 from PM_2.5_ to cognitive ability, the path b1 from cognitive ability to depression, the path a2 from PM_2.5_ to non-cognitive ability, and the path b2 from non-cognitive ability to depression. The product a1*b1 proxies the indirect effect via cognitive ability, the product a2*b2 proxies the indirect effect via non-cognitive ability, and the sum a1*b1+a2*b2 proxies the total indirect effects. The third way was Monte Carlo approach which estimated the indirect effects via cognitive ability and non-cognitive ability, respectively, and provided the ratio of the total indirect effects to the direct effects and the proportion of the total effect that was mediated.

By comparing the model without mediators and the model with mediators, it was shown that the effect of PM_2.5_ on depression was reduced in magnitude from 0.038 (path c, the total effect) to 0.028 (path c’, the direct effect). Moreover, PM_2.5_ was significantly and negatively correlated with both mediators of cognitive ability and non-cognitive ability, which were significantly and negatively correlated with depression, all these indicating there should be positive mediating effects. What’s more, since the indirect effect was still significantly distinct from zero, this was a partial mediation case.

The positive mediating effects could be demonstrated from another perspective which was the path analysis. The path a1, or path from PM_2.5_ to cognitive ability was negative at 1% significance (the second column in Table 2), and the path b1, or path cognitive ability to depression was negative at 1% significance (the fourth column in Table 2). Significantly negative paths a1 and b1 combined mean the heavier air pollution, the weaker cognitive ability, and the heavier depression. That was to say, the mediating effect via cognitive ability was positive. Similarly, the mediating effect via non-cognitive ability and the total mediating effects were also positive.

The third way to test the mediating effects was by using Monte Carlo approach, which was proposed by MacKinnon, Lockwood, and Williams [76]. Monte Carlo approach was a computer simulation test of the indirect effect, by generating a product of two independent random normal distributions of the coefficient for mediator predicted by causal variable, and the coefficient for outcome predicted by mediator. Monte Carlo approach had several advantages. One was that it didn’t need strong normality assumptions as other approaches like delta method or Sobel test, for the computer simulation resets the sample size to a large enough number to make sure that Central Limit Theorems held. The second advantage of Monte Carlo approach was that it overcame the limitation that normal theory assumption was unlikely to hold for an estimate that was the product of two normally distributed coefficients.

**Fig 3:**
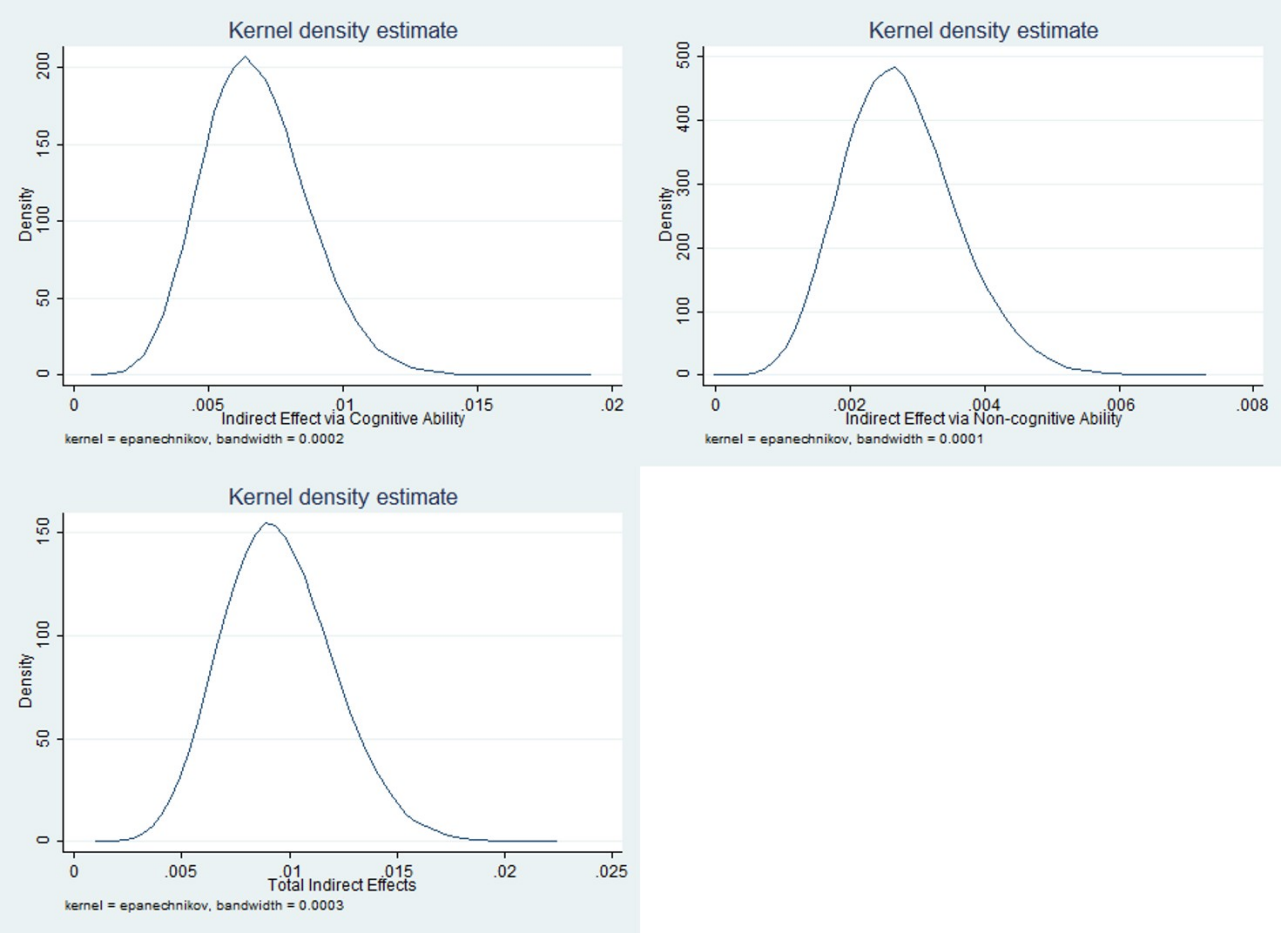
The kernel density of mediating effects of cognitive and non-cognitive ability, 2016.

**Fig 4:**
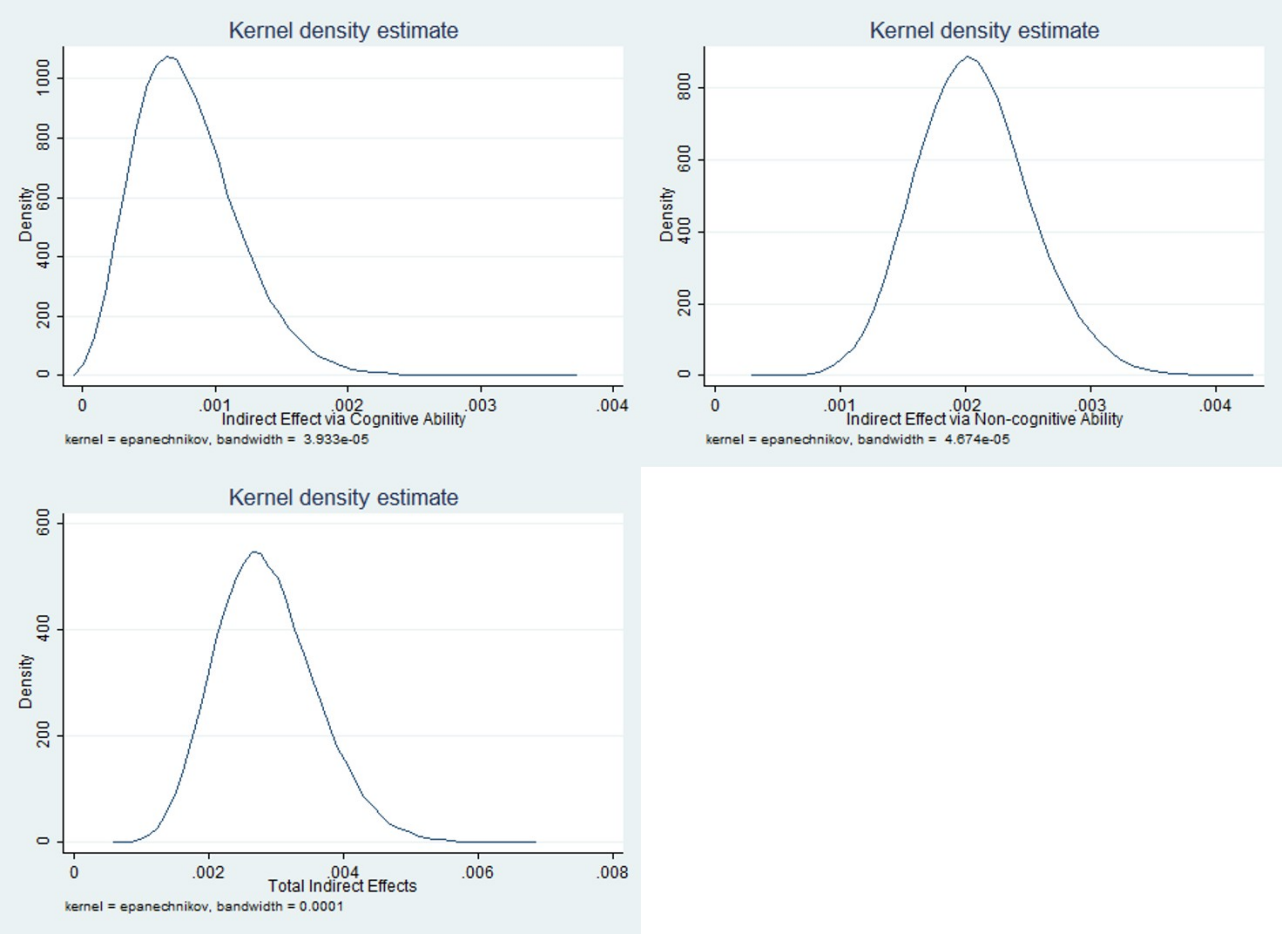
The kernel density of mediating effects of cognitive and non-cognitive ability, 2014.

The kernel density of mediating effects of cognitive and non-cognitive ability was shown in Figs 3 and 4, using data of 2016 and 2014, respectively.

From Figs 3 and 4, the Kernel density plots showed a normal distribution of the product of the coefficient of cognitive ability predicted by PM_2.5_ and the coefficient of depression predicted by cognitive ability, which was the indirect effect via cognitive ability. The positive mean values told that both the indirect effect via cognitive ability and the indirect effect via non-cognitive ability were significantly positive.

Table 6 shows the estimation of mediating effects of cognitive and non-cognitive ability using Monte Carlo approach. By estimating the product of the coefficient of cognitive ability predicted by PM_2.5_ and the coefficient of mental health predicted by cognitive ability (a1*b1), which proxied the indirect effect via cognitive ability, we obtained the mean of 0.007, the standard deviation of 0.002, and the 95% confidence interval of (0.003, 0.011). Since the confidence interval fell on the right side of zero, the indirect effect via cognitive ability was statistically significant and positive. Similarly, the indirect effect via non-cognitive ability was also statistically significant and positive. The ratio of total indirect effects to direct effect was 0.336, and the proportion of total effect that was mediated was 0.252.

**Table 6:** Estimate of mediating effects of cognitive and non-cognitive ability from monte carlo approach.

2016						
Variable	Obs	Mean	Std. Dev.	Min	Max	95% Confidence Interval
The Indirect Effect via Cognitive Ability	50000	0.007	0.002	0.001	0.019	(0.003, 0.011)
The Indirect Effect via Non-cognitive Ability	50000	0.003	0.001	0.000	0.007	(0. 001, 0.005)
The Total Indirect Effects	50000	0.010	0.003	0.001	0.022	(0.005, 0.015)
Ratio of Total Indirect Effects to Direct Effects: 0.336 Proportion of Total Effect That Is Mediated: 0.252						
2014						
Variable	Obs	Mean	Std. Dev.	Min	Max	95% Confidence Interval
The Indirect Effect via Cognitive Ability	50000	0.001	0.000	0.000	0.004	(0.000, .002)
The Indirect Effect via Non-cognitive Ability	50000	0.002	0.001	0.000	0.004	(0.001, 0.003)
The Total Indirect Effects	50000	0.003	0.001	0.001	0.007	(0.002, 0.005)
Ratio of Total Indirect Effects to Direct Effects: 0.229 Proportion of Total Effect That Is Mediated: 0.186						

## Discussion

In summary, our study found every increase of 1μg/m^3^ in PM_2.5_ deteriorating mental health by 0.038 standard deviation in 2016 wave, which showed air pollution has negative effect on mental health. By employing three different approaches as distinguishing direct effect from the total effect, path analysis, and Monte Carlo approach, we found positive mediating effects via cognitive ability and non-cognitive ability. The direct effect of air pollution on mental health could decrease to 0.028 in absolute value when considering mediation, which demonstrated the mechanism. Some literature had provided evidence that exposure to air pollution had negative effects on physical health through respiratory and cardiovascular system [13,14,17,18,24]. As a contrast, limited researches had focused on the relationship of air pollution and mental health. Using county level PM_2.5_ information from NASA and China Family Panel Survey (CFPS) with unique instrument of cognitive ability and non-cognitive ability, we have studied the relationship between air pollution and mental health together with their mediating effects.

One of the major findings was that air pollution exposure had significantly negative influence on mental health, which was consistent with the conclusions of previous studies [3,27–32,38,57]. The main biological mechanism by explaining environmental variations in nervous system could be air pollutants cross blood and brain barrier increased mental distress by different etiology and toxicity [28,29,30,32]. Since it had been supported that both cognitive ability [12,47–51] and non-cognitive ability could positively influence mental health [12,37,41,47–49] and that air pollution could damage cognitive ability [53,60–63] and non-cognitive ability [66,67], it was reasonable to propose that cognitive ability and non-cognitive ability could mediate the relationship between air pollution and mental health. And this was demonstrated by the empirical results of our study. This finding also supported previous researches that individuals had different responses to their mental health even exposed to the same stimuli such as air pollution through the mediating roles by cognitive ability and non-cognitive ability [42,43,50,51].

Our findings suggest that cognitive ability and non-cognitive ability could contribute to maintaining positive mental health was consistent with the arguments made by Davis and Humphrey [50,51] about the mediating role of coping strategies determined by cognitive ability. According to the protective factors of mental health and invest more in defensive expenditures, cognitive ability could help stay maintenance and stability, and non-cognitive ability could act as a reactive protective factor [12,42,43]. These two factors would prompt people to accurately diagnose problems and actively cope with stress in an appropriate manner [5–8]. Another mechanism related to psychology and economics by linking our findings is individual daily activities and social behaviors can be alerted from air pollution [44–46], which individuals’ cognitive and non-cognitive ability result in diverse subjective judgments with coping strategies under environmental stress.

This study made the following contributions to the literature. First of all, the study provided new evidence that air pollution could deteriorate mental health, and suggested channels to exert negative effect through damaging cognitive ability and non-cognitive ability. Secondly, using micro data from China, this study enriched evidence that cognitive and non-cognitive abilities could positively influence mental health, which was consistent with previous literature. Finally, the significance of this study was attracting more attention to environmental protection for the reason that air pollution could impair people’s mental health which was a necessary composition of human capital. Based on this national representative study of Chinese survey, our study can support the public policy and health investment on cognitive and non-cognitive abilities when coping with air pollution for better mental health. But the limitation of this study was that it did not control for the endogeneity problem caused by the probability that cognitive ability was influenced by mental health. Future research could make up for this limitation, for example, seeking for an instrument variable of cognitive ability.
